# Prevention of *Clostridium difficile* spore formation by sub-inhibitory concentrations of tigecycline and piperacillin/tazobactam

**DOI:** 10.1186/1471-2334-14-29

**Published:** 2014-01-15

**Authors:** Julian R Garneau, Louis Valiquette, Louis-Charles Fortier

**Affiliations:** 1 Département de microbiologie et d’infectiologie, Faculté de médecine et des sciences de la santé, Université de Sherbrooke, 3201 rue Jean Mignault, Sherbrooke, Québec J1E 4K8, Canada

**Keywords:** *Clostridium difficile*, Sporulation, Tigecycline, Antibiotics

## Abstract

**Background:**

Sporulation of *Clostridium difficile* during infection and persistence of spores within the gut could partly explain treatment failures and recurrence. However, the influence of antibiotics on sporulation is unclear. The objective of our study was to evaluate the impact of ciprofloxacin, metronidazole, piperacillin/tazobactam, tigecycline, and vancomycin on *C. difficile* sporulation *in vitro*.

**Methods:**

The reference strains ATCC 9689, 630, VPI 10463, and seven other clinical isolates of *C. difficile* were used, including three epidemic NAP1/027 isolates. Minimum inhibitory concentrations (MIC) were determined and sporulation was assessed after growth in the absence or presence of ≤0.5x MIC concentrations of each antibiotic.

**Results:**

All strains were sensitive to the antibiotics tested, except ribotype 027 isolates that were resistant to ciprofloxacin (MIC = 128 mg/L). Metronidazole and vancomycin generally did not significantly affect spore production in *C. difficile*, although vancomycin slightly affected sporulation of a few isolates. Ciprofloxacin inhibited sporulation of ribotype 027 isolates mainly. Interestingly, sub-MIC concentrations of piperacillin/tazobactam reduced spore formation in several isolates. However, the most striking observation was made with tigecycline, with an important reduction of spore formation in most isolates.

**Conclusions:**

The capacity of *C. difficile* to sporulate can be significantly affected by certain antibiotics. The reduced sporulation observed with tigecycline and piperacillin/tazobactam might explain why these antibiotics are generally associated with lower risk of *C. difficile* infections. In addition, the inhibition of sporulation might partly explain the apparent efficacy of tigecycline for treatment of patients with recurrent infection.

## Background

Several outbreaks caused by *Clostridium difficile* have been reported worldwide over the last decade. The emergence of an epidemic strain of *C. difficile*, NAP1/027, was associated with more severe disease, and a higher recurrence rate [1,2]. This rate ranges from 8-50% and the likelihood of recurrence increases with the number of CDI episodes [3,4], which makes the management of recurrence challenging [5-7]. Disruption of the normal intestinal microbiota is associated with a higher risk of CDI and antibiotics are generally the triggering factor [8-10]. Prolonged disruption of the intestinal microbiota by antibiotic treatment may also increase patients’ susceptibility to recurrent CDI. Recurrence can be caused by the persistence of *C. difficile* in the intestinal tract, or re-infection by the same or a different strain [5-7]. The same strain has been isolated in 50-90% of recurrence cases, which indicates that persistence of *C. difficile* spores in the intestinal tract of the patient is possibly a prerequisite to this condition [4,11].

Spores of *C. difficile* are highly resistant to harsh environments and household disinfectants and are likely responsible for efficient dissemination of *C. difficile* in hospital settings [12,13]. In addition, they are resistant to all known antibiotics including metronidazole (MTZ) and vancomycin (VAN) [14]. Some studies suggested that epidemic strains of *C. difficile* sporulate more efficiently and to higher levels than non epidemic strains, which might explain why epidemic strains disseminate easily in hospitals [12,13], but this hypothesis is a matter of debate [15,16].

Early upon infection, *C. difficile* is capable of forming spores, as suggested by the induction of sporulation-associated gene transcription as soon as 8 h post-infection [17]. However, the factors that affect this process are not well known. Previous reports suggested that sub-inhibitory (sub-MIC) concentrations of certain antibiotics can trigger *C. difficile* sporulation *in vitro*[18,19]. More recently, sub-MIC concentrations of fidaxomicin were shown to inhibit spore formation and toxin production in *C. difficile*[20,21]. This suggests that antibiotics can potentially influence the number of spores that are formed during CDI and as such, directly impact the treatment outcome and the risk of recurrence.

The objective of this study was to determine the impact of sub-MIC concentrations of 5 antibiotics on sporulation of 10 different isolates of *C. difficile in vitro*. For this, we used a microscopic method to count vegetative cells and spores present within colonies growing on agar, as well as a classical spore recovery assay after growth and sporulation in broth cultures.

## Methods

### Bacterial strains and growth conditions

*C. difficile* reference strains ATCC 9689, ATCC 43255 (VPI 10463) and 630 were used along with 7 other clinical isolates that were purified from feces after alcohol shock and growth on CDMN selective agar (*Clostridium difficile* agar base supplemented with moxalactam and norfloxacin) (Oxoid, Canada). Feces were obtained from patients recruited during a non-outbreak period at the Centre Hospitalier Universitaire de Sherbrooke in the province of Quebec, Canada. The institutional review board of the CHUS had approved our study protocol and informed consent was obtained from all patients. The identity of presumptive *C. difficile* colonies was confirmed by amplifying by PCR the triose phosphate isomerase gene (*tpi*) of *C. difficile*, as described before [16,22]. *C. difficile* was grown at 37°C in an anaerobic chamber (Coy Laboratories, USA). Bacteria were routinely grown in brain heart infusion broth (BHI) (Difco), BHI supplemented with 0.1% taurocholate and 1 mM glycine (BHI-tag) to favor spore germination, or in tryptose yeast extract broth or agar (TY) (3% tryptose (Oxoid, Canada), 2% yeast extract (BioShop, Canada)). All media were pre-reduced overnight prior to use.

### Molecular typing

Genomic DNA was extracted from 1.5 mL overnight cultures in BHI broth using the Bacteria genomicPrep kit (GE Healthcare, Canada). PCR ribotyping, tandem repeat sequence typing (TRST), detection of *tcdA*, *tcdB*, *cdtA* and *cdtB*, as well as sequencing of the *tcdC* gene was done as described before using PCR primers listed elsewhere [16].

### MIC determination

Minimum inhibitory concentrations (MIC) for MTZ (Sigma), VAN (Sigma), ciprofloxacin (CIP) (Sigma), piperacillin/tazobactam (TZP) (Sandoz), and tigecycline (TIGE) (Pfizer) were determined by the agar dilution method and interpreted according to the Clinical and Laboratory Standards Institute (CLSI) guidelines [23]. Briefly, TY agar plates were used for susceptibility testing in order to mimic the conditions of the sporulation assays. A 10-μl sample from a log-phase culture of *C. difficile* (optical density at 600 nm = 0.5) was streaked over TY agar plates containing doubling dilutions of each antibiotic. Plates were incubated under anaerobic conditions for 48 h and MIC values were determined as the antibiotic concentration where colonies did not grow.

### Evaluation of spore formation

#### Sporulation on agar plates

For time course assays, a 10-μl sample from a log-phase *C. difficile* culture grown in BHI was streaked onto TY agar plates with or without 0.5x MIC of each antibiotic. Bacteria were then grown for 48 h and 96 h and 10 colonies of similar size were picked with a sterile swab and homogenized in 0.5 mL of 0.1x BHI broth. Note that log-phase BHI pre-cultures contain only very few spores (our unpublished observation) so using this type of inoculum greatly limits carrying over spores onto TY plates when setting up the sporulation assay. Still, in the eventuality that a few spores were carried over and inoculated onto TY plates, their number was negligible since we further analyzed growing colonies, *i.e.* bacteria that grew from isolated vegetative cells or spores that have germinated and outgrown. It is also important to note that we ensured that colonies growing in the presence of 0.5x MIC antibiotic grew to a size similar to those on the control plates without antibiotic to avoid any bias due to growth defects. The number of spores and the percentage of sporulation were calculated either after recovery of viable spores and bacteria on agar, or by a microscopic method (see below).

#### Sporulation in liquid broth cultures

Spore formation was also evaluated in broth cultures. For this, log-phase cultures in BHI were inoculated in TY broth at an initial density of 1×10^6^ colony-forming units/mL (CFU/mL). Bacteria were either grown in the absence (No ATB control) or presence of 0.5x MIC antibiotics. After 48 h or 96 h of culture, samples were analyzed for the presence of vegetative cells and spores, by either recovering viable spores and bacteria on agar, or using a microscopic method, as described below.

#### Microscopic method to evaluate spore formation

A 15-μL sample from a bacterial suspension prepared from colonies grown on agar or bacteria grown in broth cultures was deposited onto microscope slides, dried at room temperature and then fixed at 80°C for 5 min. Staining with safranin was performed before microscopic observation using an Olympus IX-81 microscope equipped with a Retiga 2000R monochrome cooled CCD camera. Images from 5 different fields were taken on each slide and were processed with Metamorph v5 (Molecular Devices, USA) and ImageJ v1.44o (National Institute of Health, USA). Vegetative cells and spores were counted and the mean ± standard error of the mean (SEM) of 3 independent biological replicates (3 × 5 images) was plotted. The percentage of spores in each image was also calculated as follows: [N_spores_ / (N_spores_ + N_vegetative cells_)]*100. For concentration range assays, the same procedure described above was used except that the percentage of spores was determined at 48 h only and the antibiotic concentrations corresponded to 0.5x, 0.25x, 0.125x and 0.0625x MIC.

#### Conventional spore recovery assay to evaluate sporulation

Bacterial suspensions prepared from colonies grown on agar or bacteria grown in broth cultures were serially diluted and plated on BHI-tag agar to determine total viable counts. In parallel, aliquots from these suspensions were treated for 1 h with ethanol (50% final concentration) and then plated on BHI-tag agar to count only spores [16]. The values of CFU/mL from three independent experiments were plotted and the number of vegetative cells was calculated by subtracting the number of spores from the total count. Controls were also done in which untreated samples were plated directly on BHI without taurocholic acid and glycine, to monitor viability of vegetative cells only (spores germinate very poorly on BHI alone).

### Statistical analysis of the data

Statistical analysis of the data was done using GraphPad Prism v6.0c (California, USA). For sporulation assays determined with the microscopic method, the mean percent sporulation in the presence of antibiotic from 3 biological replicates was compared with the corresponding control without antibiotic. The same method was used to calculate spore formation on agar after ethanol shock and spore recovery on agar. For sporulation assays in broth, the total counts of vegetative cells and spores were compared (in CFU/mL, mean of 3 biological replicates). One-way analysis of variance (ANOVA) was performed in Prism to compare each condition with the corresponding controls without antibiotic. Means were considered significantly different when *p* < 0.05.

## Results

### Strain characteristics

The *C. difficile* isolates selected for sporulation assays were all toxigenic and carried the *tcdA* and *tcdB* genes (data not shown). Overall, the isolates represented 6 different PCR ribotypes and 7 TRST types (Table 1). Three isolates (CD274, CD386 and CD390) were ribotype and TRST type 027, had the characteristic 18-bp and 1-bp deletions in the *tcdC* gene, and were binary toxin-positive. Hence, these isolates correspond to the epidemic strain that caused outbreaks in Canada recently. Isolates CD385 and CD398 (ribotype 018) were also binary toxin-positive, but they had a non-deleted *tcdC* (Table 1). CD392 and CD400 had the same ribotype (014), but were of different TRST types (014 and 065 respectively).

**Table 1:** Characteristics of the selected strains

**Strain number**	**PCR ribotype**^**a**^	**TRST**^**b**^	***tcdC***^**c**^	**Binary toxin**^**d**^
ATCC 9689	001	001	-	-
630	012	012	-	-
VPI 10463 (ATCC 43255)	037	058	-	-
CD274	027	027	+	+
CD385	018	019	-	+
CD386	027	027	+	+
CD390	027	027	+	+
CD392	014	014	-	-
CD398	018	019	-	+
CD400	014	065	-	-

^a^Ribotypes were attributed according to reference strains, except ribotype 018 that was assigned arbitrarily according to our internal database.

^b^Type numbers correspond to those published by Zaiss *et al.,*[24].

^c^Presence (+) or absence (−) of the characteristic 18-bp deletion, and 1-bp deletion at nucleotide 117.

^d^Presence (+) or absence (−) of the binary toxin genes *cdtA* and *cdtB*.

### Antibiotic susceptibility testing

Susceptibility to antibiotics was determined for all strains and results are summarized in Table 2. According to the CLSI guidelines, all isolates were susceptible to the antibiotics tested, except the three ribotype 027 isolates CD274, CD386 and CD390 that were resistant to CIP [23].

**Table 2:** Antibiotic susceptibility results for the selected strains

	**MIC (mg/L)**				
**Strain**^**a**^	**CIP**	**MTZ**	**TZP**	**VAN**	**TIGE**
CD211	4	0.1	6	1	0.016
630	4	0.1	16	1	0.032
VPI 10463	8	0.2	12	1	0.032
CD274*	128	0.2	12	1	0.016
CD385	4	0.1	16	1	0.032
CD386*	128	0.1	16	1	0.016
CD390*	128	0.2	12	1	0.016
CD392	8	0.2	16	1	0.016
CD398	4	0.1	12	1	0.016
CD400	8	0.1	16	1	0.016

^a^The *indicates a ribotype 027 isolate.

### Sporulation assays

**Figure 1:**
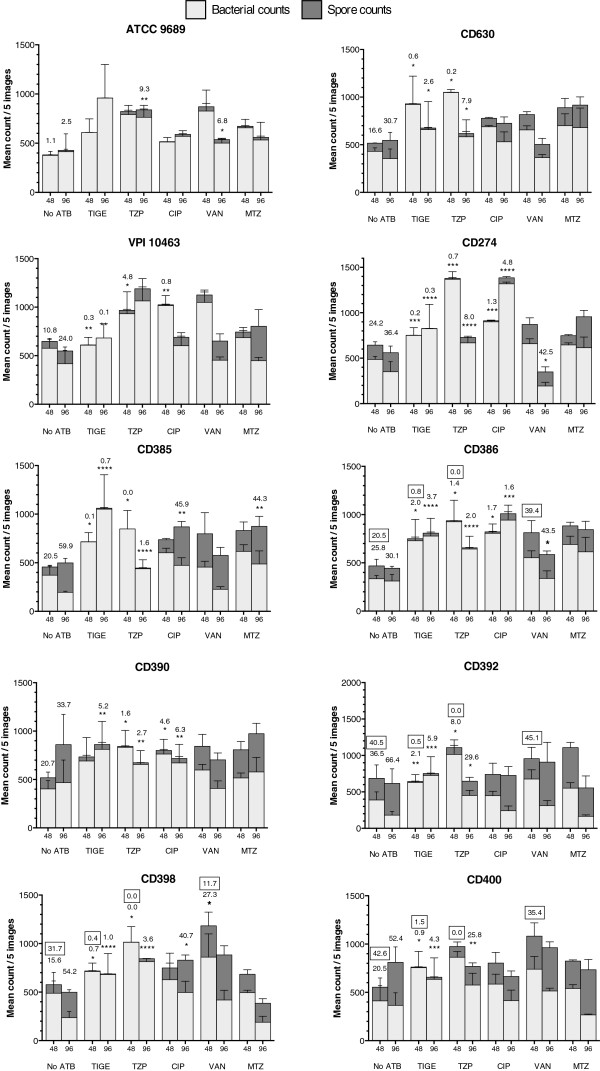
**Stacked bar graph showing the number of vegetative cells and spores in colonies grown for 48 h and 96 h on TY agar without antibiotics (No ATB) or with 0.5x MIC of TIGE, TZP, CIP, VAN, or MTZ.** The values of spore counts (dark grey bars) are stacked on top of the vegetative cell counts (lower light grey bars). The percentage of spores formed in the presence or absence of antibiotics was also calculated for each condition and compared by one-way ANOVA. When the percentage of spores was significantly lower compared to the control. For simplicity, only the % spores for the controls and the significantly different conditions are indicated above the corresponding bars. Boxed values above some of the bars represent the % spores calculated using a spore recovery assay on agar plate after ethanol shock. Values represent means ± standard error of the mean of 3 independent experiments (mean of 5 microscopy images/experiment). Statistical significance: * = p < 0.05, ** = p < 0.01, *** = p < 0.001 and **** = p < 0.0001.

The capacity of *C. difficile* isolates to sporulate in the presence of sub-MIC concentrations of antibiotic was assessed. Figure 1 is a stacked bar graph showing the mean number of vegetative cells (light grey) and spores (dark grey) counted by microscopy after 48 h and 96 h of growth in the absence or presence of 0.5x MIC of tigecycline (TIGE), piperacilin/tazobactam (TZP), ciprofloxacin (CIP), vancomycin (VAN), or metronidazole (MTZ). The % spores for each antibiotic treatment was determined and then compared with the % spores of the untreated control using ANOVA. To avoid overcrowding the graph, the % values are indicated only for the controls and the conditions where the % spores was significantly different from the controls. In the absence of antibiotic, most strains produced between ~10-35% spores at 48 h and ~25-65% at 96 h. One exception was strain ATCC 9689 that produced very few spores, even after 96 h of growth (only 2.5%). Statistical analysis of the data revealed that in the presence of 0.5x MIC of MTZ, sporulation was not significantly affected, except for strain CD385 that produced less spores at 96 h (44.3% vs 59.9% for the control). In the case of VAN, most isolates produced similar amounts of spores compared to the control group, except 4 isolates for which 0.5x MIC of VAN slightly increased the number of spores produced either after 48 h (CD398: 27.3 vs 15.6%) or 96 h (ATCC 9689: 6.8 vs 2.5%; CD274: 42.5 vs 35.4%; CD386: 43.5 vs 30.1%) but values were in the same range.

In the presence of 0.5x MIC of CIP, spore formation was significantly lower for 6/10 isolates (VPI 10463, CD274, CD385, CD386, CD390 and CD398). Interestingly the inhibition of sporulation by CIP was more pronounced at both 48 h and 96 h with strains CD274, CD386 and CD390, three ribotype 027 isolates that were resistant to CIP (Table 2). The most striking results however were observed in the presence of 0.5x MIC of TIGE and TZP, which strongly reduced spore formation in 9/10 isolates at both time points (except with TZP for CD400 at 48 h and VPI 10463 at 96 h). With these antibiotics, spore counts were very low (often <1%) in most samples as opposed to the controls or with other antibiotics for which hundreds of spores were counted (Figure 1).

In order to confirm the impact of antibiotics on sporulation and to monitor for viability of spores and vegetative cells, we performed a classical spore recovery experiment on a subset of isolates and conditions. For these assays, we included TIGE and TZP that had the strongest inhibitory effect on sporulation, and VAN that had little effect on spore formation, except with a few isolates. We selected a subset of 4 isolates of different TRST type including CD386, a ribotype 027. Colonies obtained on agar after 48 h of growth were treated with ethanol to kill vegetative cells and then serially diluted and plated on BHI-tag agar to recover spores. Untreated samples were run in parallel to recover total bacteria (spores + vegetative cells). The calculated % spores are indicated as boxed values above the corresponding bars in Figure 1. The values of % spores in the controls without antibiotic and with VAN were slightly different from the values obtained in microscopy, but they were in the same range. However, the inhibition of sporulation by TIGE and TZP that was observed in microscopy was even more evident and stronger after recovery of spores and bacteria on agar, thus confirming that TIGE and TZP really inhibit spore formation and that vegetative cells observed in microscopy were viable (counts were in the range of 10^7^-10^9^ CFU/mL).

**Figure 2:**
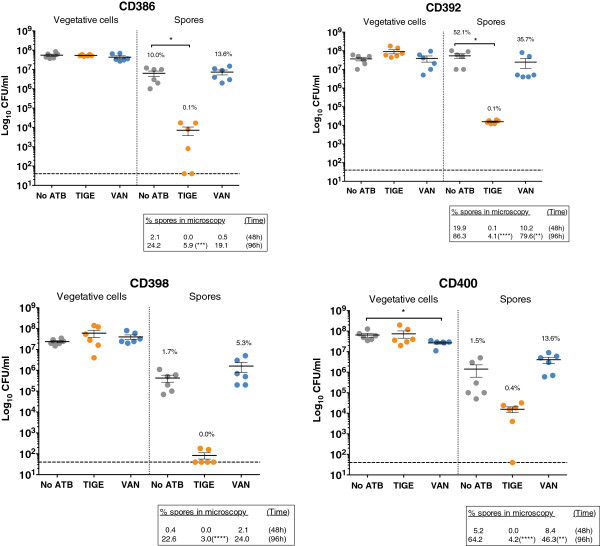
**Vegetative cells and spores growing on agar after 48 h of growth in liquid TY broth containing 0.5x MIC of TIGE (orange dots) or VAN (blue dots).** No ATB = control without antibiotic (grey dots). Dots represent the Log_10_ counts from 3 independent experiments (each done in duplicate). The dashed line indicates the limit of detection in our assay, which was 40 CFU/mL. When the spore count was below the limit of detection, the value was arbitrarily set at 40 CFU/mL. The mean values of % spores are indicated above each corresponding group for reference. The mean spore counts in the presence of antibiotics were compared with the control without antibiotics by one-way ANOVA. The boxes below each graph represent the mean % spores determined after 48 h and 96 h using the microscopic method. The values were compared by ANOVA. Statistical significance: * = p < 0.05; ** = p < 0.01; *** = p < 0.001; **** = p < 0.0001.

We also monitored the accumulation of spores over time in liquid broth in the presence or absence of antibiotics, using the same subset of isolates and TIGE and VAN for comparison. Bacteria were inoculated in TY broth and allowed to grow and sporulate for 48 h. Aliquots were removed, a fraction of which was treated with ethanol to kill vegetative cells, and serial dilutions were plated on BHI-tag agar. We also plated a series of samples on BHI alone to observe growth of vegetative cells only (spores germinate very poorly in the absence of taurocholate and glycine). As can be seen in Figure 2, the number of vegetative cells/mL that formed colonies after 48 h (calculated by subtracting the number of spores from the total count) was not significantly different in the absence or in the presence of 0.5x MIC of TIGE or VAN, thus ruling out a possible inhibitory effect of TIGE on vegetative growth. The only exception was seen with VAN and CD400 where a slightly lower number of vegetative cells could be recovered after 48 h. On average, ~10^5^ - 10^7^ spores/mL formed in the absence of antibiotic, but TIGE significantly reduced the number of spores/mL by at least 2–3 orders of magnitude in all isolates. The values of % spores calculated from the CFU/mL are indicated above the corresponding groups in Figure 2. Statistical analysis of the data using ANOVA suggests that the reduction in spore formation was not significant with CD398 and CD400 (*p* > 0.05), but a clear trend towards reduced sporulation was evident with CD398 and to a lesser extent CD400. With VAN, the amount of spores/mL was similar or slightly higher (though not statistically significant) than the control without antibiotic. We also used in parallel the microscopic method to determine the % spore after sporulation in broth culture. The values are indicated in the boxes below each corresponding graph. The number of spores/mL was low after 48 h of growth compared to the sporulation assays on agar, so we continued the incubation up to 96 h. The calculated % spores reached values similar to those in the agar assays at 96 h, and the inhibition of sporulation by TIGE was here also very strong and significant. The % spores with VAN were also lower (*p* < 0.01) with CD392 and CD400, but the inhibition in spore formation was at least 10 times stronger with TIGE. Note that we also confirmed the inhibitory effect of TZP on sporulation in broth assays as well (data not shown). Therefore, these results in broth cultures confirmed those obtained on agar, even if values did vary slightly due to the different growth conditions that might affect the dynamics of sporulation.

**Figure 3:**
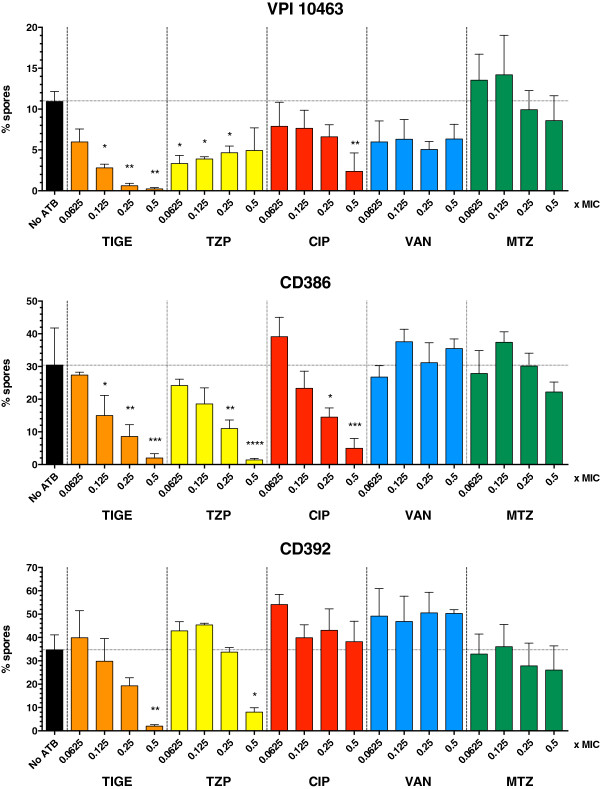
**Percentage of spores within colonies collected after 48 h of growth on TY agar containing different sub-MIC concentrations of TIGE, TZP, CIP, VAN, or MTZ.** No ATB = control without antibiotic. Values represent the means ± standard error of the mean of three independent experiments. Each condition was compared to the control without antibiotics using one-way ANOVA and asterisks indicate the means that were statistically different. Statistical significance: * = p < 0.05, ** = p < 0.01, *** = p < 0.001 and **** = p < 0.0001.

To determine if the inhibition of sporulation was dependent on the concentration of antibiotic, we performed concentration range assays on three isolates of different ribotype, including a ribotype 027 (CD386) and VPI 10463, a well-known high toxin-producing strain used in various studies including *in vivo* assays in animals [25,26]. We inoculated bacteria on agar plates containing antibiotics in the range of 0.0625-0.5x MIC and used our microscopy method to count vegetative and sporulated cells and to calculate the % spores. The results shown in Figure 3 suggest a concentration-dependent inhibition of sporulation by TIGE with all three isolates. With TZP, the inhibition of sporulation was observed at all concentrations in the case of VPI 10463 and CD386, but the inhibition did not increase proportionally to the increasing concentrations of TZP with VPI 10463 and CD392. In the later case, the inhibition was significant only at 0.5x MIC. A similar trend was seen with CIP, for which a concentration-dependent inhibition of spore formation was observed with CD386, while some inhibition was seen with VPI 10463 at 0.5x MIC. Sporulation of CD392 was not affected by CIP, which is what we expected since there was no difference in sporulation even at 0.5x MIC (Figure 1). With MTZ and VAN, there was no significant difference in the formation of spores, as also expected from the results in Figure 1.

## Discussion and conclusions

The objective of our study was to evaluate the impact of various antibiotics on the *in vitro* sporulation of a set of 10 relevant isolates of *C. difficile*. Our results strongly suggest that sub-MIC concentrations of TIGE, TZP, and to some extent CIP, have inhibitory effects on sporulation of most isolates. On the other hand, VAN slightly stimulated or inhibited spore production in a few isolates only and MTZ had no significant effect, except with one isolate where we observed a slight reduction in sporulation. Most importantly, we obtained similar results in both agar and broth sporulation assays, and using a microscopic method as well as a conventional spore recovery assay after ethanol shock and growth on agar. Therefore, the inhibition of spore formation that we observed in our study was not the consequence of a growth defect or death of vegetative cells, or poor resistance to ethanol of incompletely maturated spores. Our data rather strongly suggest that spore formation is significantly impaired. A recent study reported that *C. difficile* strains ATCC 43255 (*i.e.* VPI 10463) and UK-14 sporulated to similar levels in the absence or presence of sub-MIC of MTZ or VAN [20]. In that study, sporulation was performed over 10 days in Brucella broth and spores were counted on agar plates after heat shock. In addition, the antibiotics were added at the end of the logarithmic phase of growth and not at the time of inoculation. Under these conditions, the maximum spore counts were reached between ~24 and 96 h and remained relatively stable over the rest of the experiment [20]. Two previous studies suggested that on the contrary, MTZ and VAN strongly promoted sporulation of *C. difficile* when present at sub-MIC. Ochsner reported that sporulation of *C. difficile* strains ATCC 43596 and MB903 increased from a basal level of 10% and 17% without antibiotic to 100% after 4 days of incubation in the presence of sub-MIC concentrations of MTZ or VAN [18]. A strong increase in sporulation of strain RMA 18386 was also observed in the presence of sub-MIC of MTZ (from 0.8% to 53%) [18]. In a similar study, Mathur et al. showed that sub-MIC concentrations of MTZ and VAN also stimulated sporulation of *C. difficile* strains ATCC 43596 and PGI 1 to very high levels after 6 days of incubation (from 8.8-11% without antibiotic to 75-100% with antibiotic) [19]. In the two later studies, sporulation was assessed over 4 to 6 days on Brucella agar and the spores were counted after ethanol shock and recovery on agar plates [18,19]. We also observed increased sporulation in the presence of sub-MIC concentrations of VAN, but only with a few isolates. In addition, no difference was observed with VAN or with MTZ with most isolates. This suggests that depending on the strain of *C. difficile* under study, the impact of antibiotics on sporulation can vary and thus, a larger set of strains might be necessary to observe general trends rather than effects that might be strain-specific.

The presence of TIGE at sub-MIC concentrations strongly inhibited sporulation of 9/10 *C. difficile* isolates tested and moreover, the effect was concentration-dependent. A similar trend was also observed with TZP, although a concentration dependence was seen with only 1/3 isolates tested. Fidaxomicin was recently found to strongly inhibit the formation of spores of *C. difficile* ATCC 43255 and UK-14 when present at sub-MIC, and a lower transcription of sporulation-specific mRNAs was observed in strains CD196 and UK-1 [20]. The synthetic methionyl-tRNA synthetase inhibitor REP3123 was found to strongly inhibit sporulation of *C. difficile* ATCC 43596, MB903 and RMA 18383 in a dose-dependent manner when present at sub-MIC concentrations [18]. Similarly, the biaryl oxazolidinone molecule RBx11760 completely inhibited sporulation of *C. difficile* strains ATCC 43596 and PGI 1 at 0.5x-MIC [19]. Together, these studies also suggest that certain antibiotics have a marked inhibitory effect on sporulation of *C. difficile* when present at sub-MIC concentrations. In our study, the effect of TIGE and TZP could be observed with several isolates, thus minimizing a possible strain-specific effect. The only isolate that did not respond like the others was ATCC 9689, but the number of spores formed by this strain was already very low, making it more difficult to see inhibition of sporulation by antibiotics. On the contrary, VAN, TZP and MTZ tended to increase spore formation by this isolate.

Babakhani reported that fidaxomicin affected spore formation possibly by blocking the accumulation of *spoIIR* and *spoIIID* mRNAs, which are expressed specifically during sporulation [20]. This is thought to be due to the anti-RNA polymerase activity of fidaxomicin, but whether the inhibition applies to all bacterial genes or only to sporulation genes is unknown [20]. We noted a decrease in sporulation in the presence of CIP, especially with ribotype 027 strains. For those particular isolates, higher CIP concentrations had to be incorporated into agar plates to reach 0.5x MIC, because of their intrinsic resistance to CIP. Thus, although growth of these strains was not affected, high CIP concentrations seemed to alter sporulation somehow. Whether CIP interferes with transcription of sporulation-specific genes like fidaxomicin remains to be determined, but since CIP interferes with DNA synthesis and replication, this would not, a priori, be logical. However, a number of studies have shown that transcription of virulence-associated genes in *C. difficile* can be affected by antibiotics that are not inhibitors of transcription per se. For example, Gerber *et al.* observed that 0.5x MIC of MTZ, VAN, and linezolid increased transcription of *tcdA* and *tcdB* toxin genes in 4 different strains of *C. difficile*, including VPI 10463 [27]. Toxin gene transcription and production were also increased in high-level CIP-resistant isolates of *C. difficile*, and a dose-dependent response was observed [28]. Likewise, sub-MIC concentrations of ampicillin and clindamycin strongly increased transcription of genes coding for the colonization factors Cwp84 and the surface layer protein SlpA in NAP1/027 isolates [29]. In addition, ofloxacin and moxifloxacin increased transcription of *cwp84* and *slpA*, but only in ofloxacin and moxifloxacin-resistant isolates [29]. Altogether, current data from the literature suggest that sub-inhibitory concentrations of certain antibiotics can affect virulence-associated phenotypes and sporulation in resistant strains, in part via modulation of transcription.

It is noteworthy to mention that we observed an inhibition of sporulation with 3 different antibiotics that have different modes of action: TIGE inhibits protein synthesis, CIP interferes with DNA synthesis and replication, and TZP interferes with cell wall synthesis. A possible mechanism explaining the inhibition of sporulation is thus rather speculative at the moment, but the fact that REP3123 and RBx11760 are both protein synthesis inhibitors led Mathur to propose that the inhibitory effect on sporulation was probably due to a general inhibition of the spore coat protein synthesis [18,19]. This hypothesis may apply to TIGE as well, which is also an inhibitor of protein synthesis, but does not reconcile the results obtained with TZP and CIP. However, as mentioned above, transcription of several genes can be affected by antibiotics, including quinolones to which bacteria are already resistant [29]. Of note, bacteria grew well in the presence of sub-MIC concentrations of all antibiotics tested in our study, suggesting that the inhibition of sporulation by TZP and high CIP concentrations was not directly linked to the growth capacity or cell viability, as reported previously with other antibiotics [27,29,30]. Further studies will be necessary to elucidate the molecular mechanism by which CIP, TZP and TIGE inhibit sporulation.

The biological significance of the inhibition of sporulation by sub-inhibitory concentrations of antibiotics *in vitro* remains to be determined *in vivo*. Although fecal concentrations of TIGE are generally much higher than the MIC for *C. difficile* (range of 3.0-14.1 μg/g feces) [31], it is possible that sub-MIC concentrations could occur early at the beginning, or at the very end of the antibiotic treatment, during which period sporulation could be affected. A recent transcriptomic analysis in mice mono-colonized with *C. difficile* revealed that transcription of sporulation-associated genes was upregulated as soon as 8 h post infection, therefore suggesting that spores are formed early *in vivo*. It is thus reasonable to suggest that inhibiting sporulation early after infection could possibly reduce the risk of relapse due to persistence of spores in the gut. Recent studies also suggest that *C. difficile* can form biofilms [32,33], and it was suggested that biofilms could possibly protect *C. difficile* from antibiotics, creating an environment where sub-MIC concentrations of antibiotics could be present [34]. In the case of fidaxomicin, high fecal concentrations, way above the MIC, were also reported and recent experimental data suggest that the better performance of fidaxomicin compared to vancomycin could possibly be due to inhibition of sporulation and toxin production, as determined in the presence sub-MIC concentrations of the antibiotic [20,21]. Case reports have shown that TIGE, alone or in combination with other antibiotics, was able to cure patients with recurrent CDI that were refractory to MTZ and/or VAN [35,39]. The exact reason why TIGE seems effective in treating recurrent CDI remains to be elucidated and warrants further investigations, but our study suggests that the inhibition of sporulation could be one possible explanation, like for fidaxomicin [20].

## Competing interests

L.V. has served on advisory boards for Oryx, Iroko, Abbott and Wyeth, and has received compensation to conduct clinical trials involving antibacterials from Genzyme, Wyeth, Pfizer, BioCryst, Trius, Cempra, Optimer and Arpida. All other authors have no competing interest to declare.

## Authors’ contributions

Conceived and designed the experiments: LV, LCF. Performed the experiments: JRG. Analyzed the data: JRG, LV, LCF. Wrote the paper: LV, LCF. All authors read and approved the final manuscript.

## Pre-publication history

The pre-publication history for this paper can be accessed here:

http://www.biomedcentral.com/1471-2334/14/29/prepub
